# Prognostic significance of proline, glutamic acid, leucine rich protein 1 (PELP1) in triple-negative breast cancer: a retrospective study on 129 cases

**DOI:** 10.1186/s12885-015-1694-y

**Published:** 2015-10-15

**Authors:** Yanzhi Zhang, Jiali Dai, Keely M. McNamara, Bing Bai, Mumu Shi, Monica S. M. Chan, Ming Liu, Hironobu Sasano, Xiuli Wang, Xiaolei Li, Lijuan Liu, Ying Ma, Shuwen Cao, Yanchun Xing, Baoshan Zhao, Yinli Song, Lin Wang

**Affiliations:** 1 Department of Pathology, Harbin Medical University-Daqing, No. 39 Xinyang Road, Hi-Tech Zone, Daqing, Heilongjiang China; 2 Department of Pathology, Tohoku University School of Medicine, Sendai, Japan; 3 Department of Histology and Biology, Harbin Medical University-Daqing, Daqing, China; 4 Department of Pathology, The Fifth Affiliated Hospital of Harbin Medical University, Daqing, China; 5 Department of Pathology, Daqing Oilfield General Hospital, Daqing, China; 6 Department of Pathology, Daqing Longnan Hospital, Daqing, China

**Keywords:** Proline, Glutamic acid, Leucine rich protein 1, Triple negative breast cancer, Prognosis, Immunohistochemistry

## Abstract

**Background:**

Triple-negative breast cancer (TNBC) is associated with an aggressive clinical course due to the lack of therapeutic targets. Therefore, identifying reliable prognostic biomarkers and novel therapeutic targets for patients with TNBC is required. Proline, glutamic acid, leucine rich protein 1 (PELP1) is a novel steroidal receptor co-regulator, functioning as an oncogene and its expression is maintained in estrogen receptor (ER) negative breast cancers. PELP1 has been proposed as a prognostic biomarker in hormone-related cancers, including luminal-type breast cancers, but its significance in TNBC has not been studied.

**Methods:**

PELP1 immunoreactivity was evaluated using immunohistochemistry in 129 patients with TNBC. Results were correlated with clinicopathological variables including patient’s age, tumor size, lymph node stage, tumor grade, clinical stage, histological type, Ki-67 LI, as well as clinical outcome of the patients, including disease-free survival (DFS) and overall survival (OS).

**Results:**

PELP1 was localized predominantly in the nuclei of carcinoma cells in TNBC. With the exception of a positive correlation between PELP1 protein expression and lymph node stage (*p* = 0.027), no significant associations between PELP1 protein expression and other clinicopathological variables, including DFS and OS, were found. However, when PELP1 and Ki-67 LI were grouped together, we found that patients in the PELP1/Ki-67 double high group (n = 48) demonstrated significantly reduced DFS (*p* = 0.005, log rank test) and OS (*p* = 0.002, log rank test) than others (n = 81). Multivariable analysis supported PELP1/Ki-67 double high expression as an independent prognostic factor in patients with TNBC, with an adjusted hazard ratio of 2.020 for recurrence (95 % CL, 1.022–3.990; *p* = 0.043) and of 2.380 for death (95 % CL, 1.138–4.978; *p* = 0.021).

**Conclusions:**

We found that evaluating both PELP1 and Ki-67 expression in TNBC could enhance the prognostic sensitivity of the two biomarkers. Therefore, we propose that PELP1/Ki-67 double high expression in tumors is an independent prognostic factor for predicting a poor outcome for patients with TNBC.

## Background

Breast cancer is a heterogeneous disease that harbors various genetic alterations allowing it to be classified into distinct molecular subtypes that respond differently to therapy and are associated with various clinical outcomes [1]. Triple-negative breast cancer (TNBC), one of the three IHC-defined subtypes routinely assessed in clinical practice, is characterized by the lack of expression of estrogen receptor alpha (ERα) and progesterone receptor (PR), as well as non-amplified levels of human epidermal growth factor receptor 2 (HER-2) in carcinoma cells. The TNBC subtype is generally associated with an aggressive clinical course and worse prognosis due to the lack of available targeted therapeutic measures, such as aromatase inhibitors or trastuzumab treatment [2].

Traditional prognostic parameters used in the assessment of breast cancer outcomes, such as histological type, lymph node stage, and Nottingham prognosis index, may influence the prognosis of individual TNBC patients. However, as a group, TNBC patients with similar prognostic parameters often experience rather different clinical outcomes [3]. Therefore, it has become important to identify new prognostic biomarkers for TNBC patients. Several factors such as the mesenchymal-to-epithelial transition factor [4], Lewis X [5], and breast cancer type 1 susceptibility protein (BRCA1) [6] have been proposed as prognostic markers for TNBC patients, but their predictive significance is uncertain.

Proline, glutamic acid, leucine rich protein 1 (PELP1; also known as a modulator of non-genomic activity of the estrogen receptor) is a novel steroidal receptor co-regulator. Of great interest, in contrast to other steroidal receptor co-regulators, PELP1 is involved in both genomic and non-genomic functions of steroidal signaling and exhibits oncogenic properties [7]. Specifically, PELP1 overexpression has been reported to induce the malignant transformation of normal cells, accelerate cell cycle progression, promote tumor cell proliferation, and enhance the migration and invasion of tumor cells [8]. PELP1 was initially identified as a co-regulator of ERα, but its expression is also remarkably high in ERα-negative breast cancers [9, 10]. Additionally, reduction of PELP1 in ERα-negative breast cancer cell lines reduced proliferation and tumor metastasis, suggesting a role for PELP1 in tumor progression [10]. Therefore, PELP1 is postulated to function independently of ERα in breast carcinoma cells.

Several studies also proposed PELP1 as a prognostic biomarker in hormone-related cancers, including endometrial [11], ovarian [12], colorectal [13], and luminal-type breast carcinomas [14]. However, the predictive value of PELP1 in TNBC has remained unclear. Therefore, in this study, we retrospectively assessed PELP1 immunoreactivity in 129 patients with TNBC, and correlated the status of PELP1 independently, or in combination with other clinicopathological variables, to the outcomes of the patients.

## Methods

### Patients

TNBC was defined as breast carcinomas with negative expression of ERα (positive tumor nuclei <1 % on immunohistochemistry), PR (positive tumor nuclei <1 % on immunohistochemistry), and HER-2 expression (HercepTest score <2 on immunohistochemistry or HercepTest score = 2 on immunohistochemistry with HER2/CEP17 ratio <2.2 by fluorescence in situ hybridization). A total of 159 cases of patients diagnosed as TNBC at The Fifth Affiliated Hospital of Harbin Medical University, Daqing Oilfield General Hospital and Daqing Longnan Hospital were collected. Clinical information (including patient’s age, tumor size, lymph node stage, tumor grade, histological type, clinical stage), pathological biomarkers information [including status of ER, PR, HER-2, and Ki-67 label index (Ki-67 LI) (Ki-67 LI was defined as the percentage of tumor cells showed nuclear immunoreactivity with MIB-1)], and primary treatment (including surgery, chemotherapy, and radiotherapy) were retrieved from the medical records at these three institutions. Twelve patients were excluded from the study cohort because of gender (male), or acceptance of neo-adjuvant chemotherapy. The pathological slides of the remaining 147 patients were reviewed by two of the authors (JLD and BSZ.) blinded to the clinical and follow-up data. Subsequently, 18 were excluded for discordance between the reviewers, leaving 129 patients in the study. These cases consisted of 49 patients diagnosed at The Fifth Affiliated Hospital of Harbin Medical University from 2001 to 2011, 45 cases diagnosed at Daqing Longnan Hospital from 2002 to 2010, and 35 patients diagnosed at Daqing Oilfield General Hospital from 2004 to 2011. Formalin fixed, paraffin-embedded surgical excisional tissue blocks from each selected patient were collected for detecting PELP1 protein expression. The protocol of this study is in accordance with the Helsinki Declaration and was approved by the institutional review board of Harbin Medical University. The approvals for this study were obtained from all the three hospitals involved, written informed consent was obtained from all participants.

### Immunohistochemistry (IHC)

For immunohistochemistry, all samples were prepared as 5-μm-thick serial sections mounted on glass slides. Slides were deparaffinized with xylene and rehydrated on alcohol gradients. Endogenetic peroxidase was blocked with 3 % hydrogen peroxide-methanol for 30 minutes. For antigen retrieval, specimens were heated for 15 minutes in 10-mM citrate buffer (pH6.0) by microwaving (500 W). Polyclonal antibody against PELP1 (Cat. IHC-00013, Bethyl Laboratories, Inc. Montgomery, AL, USA) was applied at an optimized dilution of 1:200 at 4 °C overnight. Real Envision Detection system (DAKO, Denmark) was used instead of the traditional secondary antibody, sections were visualized with the chromogen DAB and counterstained with hematoxylin. The specificity of the PELP1 antibody was checked by western blot using a standard protocol. For quality control, a breast cancer specimen with definite PELP1 protein expression was used as a positive control, while a negative control was performed by omitting the primary antibody and substituting it with antibody dilution buffer (DAKO, Denmark).

The PELP1 immunoreactivity was evaluated independently by two of the authors (ML and SWC), both of whom were blinded to the clinical and follow up data of the samples. H-score was used to quantify the immunoreactivity of PELP1, as previously described [14]. In brief, PELP1 staining intensity was scored as 0, 1, 2, and 3, and the percentage of positive cells was determined for each score to produce a final score in the range 0–300. The optimized cutoff points in the Habashy et al. study were also adopted for this study, the cut-off points were defined using the X-tile program, and the immunoreactivity of PELP1 were classified into negative (H-score <5), moderate (5 ≤ H-score <170) and strong (170 ≤ H-score) [14].

### Statistical analysis

Statistical analysis was performed using SPSS 17.0 statistical software (Chicago, IL, USA). Association between PELP1 protein expression and different clinicopathological variables was studied using the chi-square test. The primary endpoint of this study was disease-free survival (DFS), and the second endpoint was overall survival (OS). DFS was defined as the period from the date of primary surgery to the date of diagnosis as local or distant recurrence, OS was defined as the period between the date of primary surgery and the date of death (from any cause). Univariable survival curves were estimated by the Kaplan-Meier method and tested with the log rank test. Multivariable analysis for DFS and OS were performed using the Cox proportional hazards regression model (Enter method). *p* < 0.05 was considered significant.

## Results

### Patient information

The median age of the patients at the time of their first surgery was 50 years (range 26–75). The median follow-up was 40 months (range 2–87). At the end of this study, 29.4 % (38/129) of the patients experienced local/distant recurrence, and 24.8 % (32/129) died. The clinicopathological variables of the patients are summarized in Table 1. For all collected variables, no significant difference was found among the cohorts from The Fifth Affiliated Hospital of Harbin Medical University, Daqing Longnan Hospital, and Daqing Oilfield General Hospital (data not shown).

**Table 1:** Patient clinical pathological variables

Clinical pathological variables	number
Age (years)	
≤50	69 (53.5 %)
>50	60 (46.5 %)
Tumor size (cm)^a^	
≤2	31 (24.0 %)
>2, ≤5	74 (57.4 %)
>5	22 (17.1 %)
Unavailable	2 (1.6 %)
Lymph node stage	
negative	65 (50.4 %)
positive	64 (49.6 %)
Grade	
G1	24 (18.6 %)
G2	30 (23.3 %)
G3	75 (58.1 %)
Clinical stage^a^	
I	19 (14.7 %)
II	62 (48.1 %)
III and IV	46 (35.7 %)
Unavailable	2 (1.6 %)
Histological type	
IDC	101 (78.3 %)
ILC	18 (14.0 %)
Others	10 (7.8 %)
Ki-67 LI	
Low (≤14 %)	39 (30.2 %)
High (>14 %)	90 (69.8 %)
Chemotherapy	
AC	45 (34.9 %)
AC-T	72 (55.8 %)
Others	10 (7.8 %)
None	2 (1.6 %)
Radiotherapy	
No	67 (51.9 %)
Yes	62 (48.1 %)
Cohort	
FAHHMU	49 (38.0 %)
DLH	45 (34.9 %)
DOGH	35 (27.1 %)

Abbreviations: LN, lymph node; IDC, invasive ductal carcinoma; ILC, invasive lobular carcinoma; Ki-67 LI, Ki-67 label index; AC, Adriamycin/Cyclophosphamide; AC-T, Adriamycin/Cyclophosphamide-Taxol; FAHHMU, The Fifth Affiliated Hospital of Harbin Medical University; DLG, Daqing Longnan Hospital; DOGH, Daqing Oilfield General Hospital

Note: ^a^for the variable, data for two cases are unavailable from medical records

### PELP1 protein expression

**Fig. 1:**
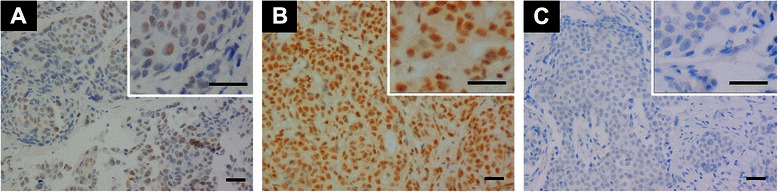
Immunohistochemical staining of PELP1 in TNBC. Positive immunostaining of PELP1 mainly distributed in nuclei of tumor cells, no cytoplasmic staining was found (**a**, **b**). Low grade lymph node stage TNBC showed weak PELP1 nuclear expression (**a**), High grade lymph node stage TNBC showed strong PELP1 nuclear expression (**b**). PELP1 nuclear staining was absent in negative control (**c**). Bar = 50 μm.

PELP1 protein immunostaining was exclusively localized to the nuclei of tumor cells, with no cytoplasmic staining observed in any sample in this cohort. In some cases, weak nuclear immunostaining of PELP1 could also be observed in ductal epithelial cells and fibroblasts of the surrounding normal tissues (Fig. 1). Among our TNBC cohort, the lowest H-score of PELP1 was 12. Consequently, none of the samples were classified into the negative group, 45.7 % (59/129) of the cases were classified into the moderate group and 54.3 % (70/129) were classified into the strong group. Thus, two groupings emerged: a PELP low group and a PELP high group, corresponding to the Habashy et al. moderate and strong classifications, respectively [14].

### Correlation of PELP1 protein expression with other clinicopathological variables

The expression of PELP1 in TNBC was compared to clinicopathological variables including patient’s age, tumor size, lymph node stage, tumor grade, clinical stage, histological type, Ki-67 LI, and primary treatment to see if there were correlations between PELP1 and these variables. The cut-off value for each of these variables was a standardized value that was in line with previous publications [15]. With the exception of a positive correlation between PELP1 protein expression and lymph node stage (*p* = 0.027), no significant association between PELP1 protein expression and other clinicopathological variables was found (Table 2).

**Table 2:** Correlation between PELP1 protein expression and clinicopathological variables in patients with TNBC

Variables	*n*	Status of PELP1 protein expression		*P*-value
		low	high	
Age (years)				
≤50	69	31 (44.9 %)	38 (55.1 %)	0.843
>50	60	28 (46.7 %)	32 (53.3 %)	
Tumor size (cm)^a^				
≤2	31	13 (41.9 %)	18 (58.1 %)	0.635
>2, ≤5	74	33 (44.6 %)	41 (55.4 %)	
>5	22	12(54.5 %)	10 (45.5 %)	
Lymph node stage				
negative	65	36 (55.4 %)	29 (44.6 %)	0.027
positive	64	23 (35.9 %)	41 (64.1 %)	
Grade				
G1	24	13 (54.2 %)	11 (45.8 %)	0.612
G2	30	14 (46.7 %)	16 (53.3 %)	
G3	75	32 (42.7 %)	43 (57.3 %)	
Clinical stage^a^				
I	19	11 (57.9 %)	8 (42.1 %)	0.374
II	62	29 (46.8 %)	33 (53.2 %)	
III and IV	46	18 (39.1 %)	28 (60.9 %)	
Histological type				
IDC	101	45 (44.6 %)	56 (55.4 %)	0.250
ILC	18	11 (61.1 %)	7 (38.9 %)	
Others	10	3 (30.0 %)	7 (70.0 %)	
Ki-67 LI				
Low (≤14 %)	39	17 (43.6 %)	22 (56.4 %)	0.747
High (>14 %)	90	42 (46.7 %)	48 (53.3 %)	
Chemotherapy				
AC	45	22 (48.9 %)	23 (51.1 %)	0.945
AC-T	72	32 (44.4 %)	40 (55.6 %)	
Others	10	4 (40.0 %)	6 (60.0 %)	
None	2	1 (50.0 %)	1 (50.0 %)	
Radiotherapy				
No	67	30 (44.8 %)	37 (55.2 %)	0.820
Yes	62	29 (46.8 %)	33 (53.2 %)	
Cohort				
FAHHMU	49	22 (44.9 %)	27 (55.1 %)	0.820
DLH	45	21 (46.7 %)	24 (53.5 %)	
DOGH	35	16 (45.7 %)	19 (54.3 %)	

Abbreviations: LN, lymph node; IDC, invasive ductal carcinoma; ILC, invasive lobular carcinoma; Ki-67 LI, Ki-67 label index; AC, Adriamycin/Cyclophosphamide; AC-T, Adriamycin/Cyclophosphamide-Taxol; FAHHMU, The Fifth Affiliated Hospital of Harbin Medical University; DOGH, Daqing Oilfield General Hospital

Note: ^a^for the variable, data for two cases are unavailable from medical records

### Clinicopathological variables and patient outcome

**Fig. 2:**
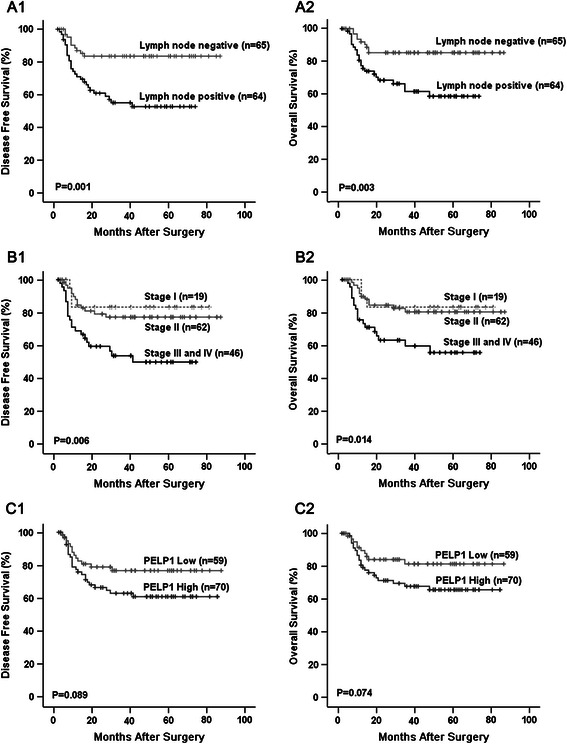
Clinicopathological variables and outcomes of patients with TNBC. Kaplan–Meier survival curve showed that TNBC patients with positive lymph node metastasis had significantly reduced DFS (**a1**) and OS (**a2**); TNBC patients in stage III and IV also demonstrated significantly reduced DFS (**b1**) and OS (**b2**); PELP1 was not associated with DFS or OS in TNBC patients when observed independently, although patients in the high PELP1 group demonstrated a trend of reduced DFS (**c1**) and OS (**c2**), compared with those in the low PELP1 group.

Kaplan–Meier survival analysis revealed that patients with higher lymph node stage or clinical stage have significantly reduced DFS and OS (Fig. 2a, b). No significant association between the other observed variables and patient survival were found, including the status of PELP1 (Table 3), although patients in the high PELP1 group demonstrated a trend of reduced DFS and OS, compared with those in the low PELP1 group (Fig. 2c).

**Table 3:** Univariate analysis of DFS and OS according to clinicopathological variables

Variables		DFS		OS	
		*χ* ^2^	*P*-value	*χ* ^2^	*P*-value
Age (years)	≤50 *vs.* >50	0.058	0.810	0.436	0.509
Tumor size (cm)^a^	≤2 *vs.* >2, ≤5 *vs.* >5	2.515	0.284	2.608	0.271
Lymph node stage	negative *vs*. positive	11.706	0.001	8.803	0.003
Grade	G1 *vs.* G2 *vs.* G3	4.764	0.092	3.204	0.201
Clinical stage^a^	I *vs*. II *vs*. III and IV	10.343	0.006	8.576	0.014
Histological type	IDC *vs.* ILC *vs.* Others	0.588	0.745	0.883	0.643
Ki-67 LI	Low (≤14 %) *vs.* High (>14 %)	1.974	0.160	2.709	0.100
Chemotherapy	AC *vs.* AC-T *vs.* Others vs. None	0.664	0.882	1.194	0.754
Radiotherapy	No *vs.* Yes	0.091	0.763	0.002	0.963
PELP1 status	Low *vs*. High	2.887	0.089	3.182	0.074

Abbreviations: LN, lymph node; IDC, invasive ductal carcinoma; ILC, invasive lobular carcinoma; Ki-67 LI, Ki-67 label index; AC, Adriamycin/Cyclophosphamide; AC-T, Adriamycin/Cyclophosphamide-Taxol; DFS, disease-free survival; OS, overall survival

Note: ^a^for the variable, data for two cases are unavailable from medical records

### PELP1 protein expression and patient outcome in TNBC subgroups

**Fig. 3:**
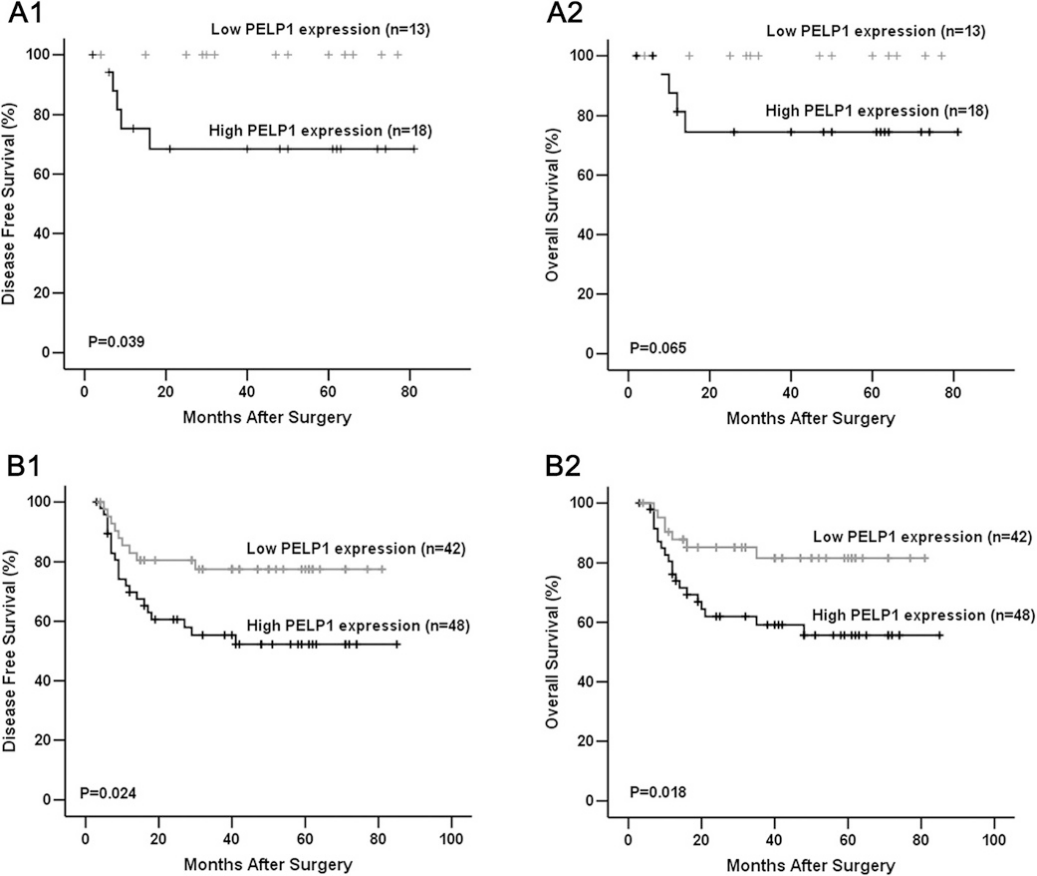
PELP1 protein expression and patients’ outcome in subgroups of TNBC. Kaplan–Meier survival curve showed that, in the tumor size ≤ 2 cm subgroup, patients with high PELP1 expression had significantly shorter DFS (**a1**); in the high Ki-67 LI subgroups, patients with high PELP1 expression have significantly shorter DFS (**b1**) and OS (**b2**).

To further explore the prognostic significance of PELP1 in TNBC, we subgrouped the patients according to age, tumor size, lymph node stage, tumor grade, histological type, clinical stage, Ki-67 LI, chemotherapy, and radiotherapy, and correlations between PELP1 protein expression and patient’s outcome in the different subgroups were examined using Kaplan–Meier analysis. In the subgroup with tumor size ≤ 2 cm, patients with high PELP1 protein expression showed significantly shorter DFS compared with those with low PELP1 expression (Fig. 3a). In the subgroup with high Ki-67 LI (>14 %), both DFS and OS of patients with high PELP1 expression were significantly shorter than those with low PELP1 expression (Fig. 3b). No significant correlation between PELP1 expression and patient’s outcome was found in any other subgroup (Table 4).

**Table 4:** Univariate analysis of DFS and OS according to PELP1 protein expression in different subgroups

Variables	Subgroup	DFS		OS	
		*χ* ^2^	*P*-value	*χ* ^2^	*P*-value
Age (years)					
	≤50	1.636	0.201	1.759	0.185
	>50	1.183	0.277	1.246	0.264
Tumor size (cm)^a^					
	≤2	4.274	0.039	3.398	0.065
	>2, ≤5	0.441	0.507	0.813	0.367
	>5	1.936	0.164	1.134	0.284
Lymph node stage					
	negative	0.251	0.617	0.008	0.927
	positive	0.770	0.380	1.974	0.160
Grade					
	G1	1.864	0.172	0.688	0.407
	G2	2.369	0.124	2.327	0.127
	G3	0.188	0.665	0.461	0.497
Clinical stage^a^					
	I	1.231	0.267	1.250	0.264
	II	0.258	0.612	0.009	0.926
	III and IV	1.814	0.178	3.220	0.073
Histological type					
	IDC	1.278	0.258	1.399	0.237
	ILC	1.780	0.182	1.591	0.207
	Others	0.928	0.335	0.928	0.335
Ki-67 LI					
	Low (≤14 %)	0.148	0.700	0.161	0.688
	High (>14 %)	5.069	0.024	5.559	0.018
Chemotherapy					
	AC	1.144	0.285	1.192	0.275
	AC-T	1.910	0.157	1.871	0.171
	Others	0.000	0.994	0.500	0.480
Radiotherapy					
	No	2.806	0.094	3.262	0.071
	Yes	0.460	0.498	0.488	0.485

Abbreviations: LN, lymph node; IDC, invasive ductal carcinoma; ILC, invasive lobular carcinoma; Ki-67 LI, Ki-67 label index; AC, Adriamycin/Cyclophosphamide; AC-T, Adriamycin/Cyclophosphamide-Taxol; DFS, disease-free survival; OS, overall survival

Note: ^a^for the variable, data for two cases are unavailable from medical records

### Combining PELP1 status and Ki-67 LI as a prognostic biomarker

**Fig. 4:**
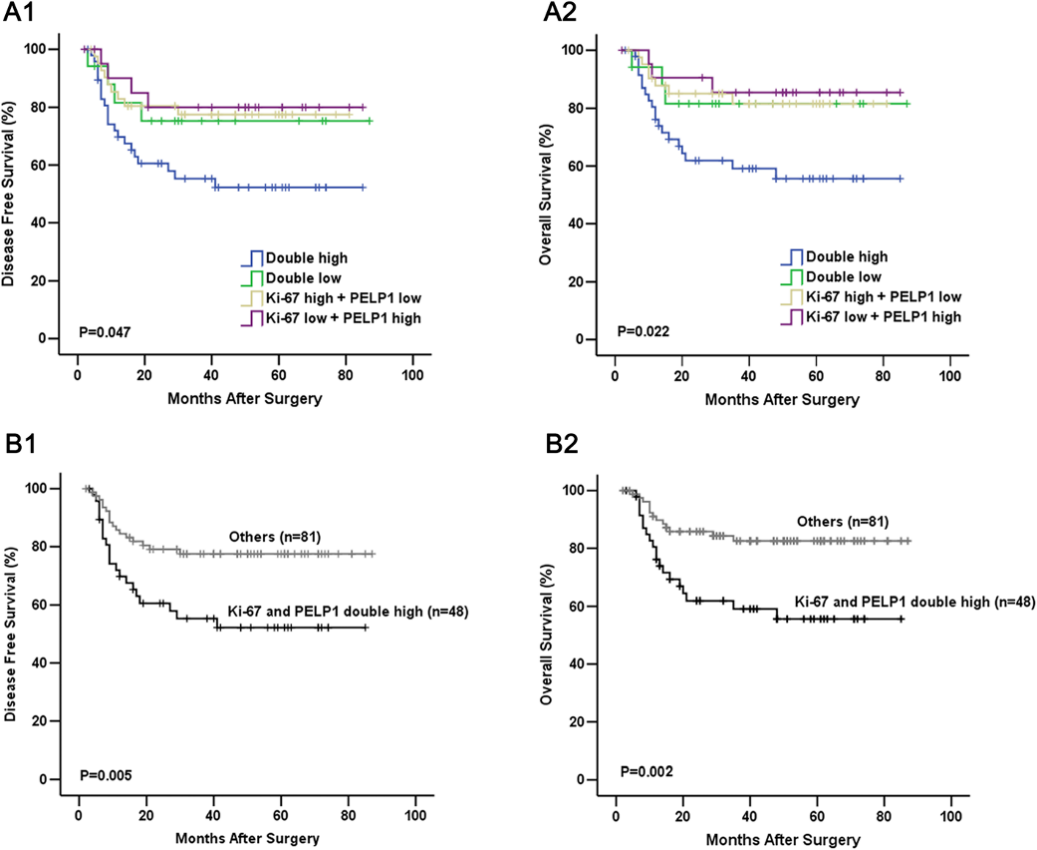
Combining PELP1 status and Ki-67 LI as a prognostic biological marker. Kaplan–Meier survival curve showed that, combination of PELP1 status with Ki-67 status was significantly correlated with DFS (**a1**) and OS (**a2**) in patients with TNBC; patients with TNBC in PELP1/Ki-67 double high group had significantly reduced DFS (**b1**) and OS (**b2**) compared with others.

Considering that we found a significant correlation between PELP1 status and DFS, as well as between PELP1 status and OS, but only in the high Ki-67 LI subgroup, we further examined whether combining PELP1 status and Ki-67 LI can be used as a prognostic biomarker for the whole TNBC cohort. The patients were subgrouped into four groups according to PELP1 status and Ki-67 LI: PELP1/Ki-67 double low, PELP1 low/Ki-67 high, PELP1 high/ Ki-67 low, and PELP1/Ki-67 double high groups, and submitted for univariate survival analysis. For the four groups, Kaplan–Meier analysis showed a significant difference related to DFS (*p* = 0.047) and OS (*p* = 0.022). Additionally, this difference mainly existed between PELP1/Ki-67 double high group and the others (Fig. 4a). Subsequent analysis revealed that patients in the PELP1/Ki-67 double high group (n = 48) had significantly reduced DFS (*p* = 0.005, log rank test) and OS (*p* = 0.002, log rank test) than others (n = 81) (Fig. 4b).

### Multivariable analysis

The independent effect of PELP1/ Ki-67 double high expression on DFS and OS was assessed using a multivariable Cox proportional hazards regression model, adjusted for patient age, tumor size, lymph node stage, tumor grade, and histological type. The analysis supported PELP1/Ki-67 double high expression as an independent prognostic factor in patients with TNBC, with an adjusted hazard ratio (HR) of 2.020 for recurrence (95 % CL, 1.022–3.990; *p* = 0.043) and of 2.380 for death (95 % CL, 1.138–4.978; *p* = 0.021) (Table 5).

**Table 5:** Multivariate analysis of DFS and OS according to clinical pathological variables

Variables		DFS			OS		
		HR	95 % CL	*P*-value	HR	95 % CL	*P*-value
Age (years)	≤50 *vs.* >50	0.786	0.404-1.527	0.477	0.698	0.340-1.432	0.327
Tumor size (cm)^a^	≤2 *vs.* >2, ≤5 *vs.* >5	1.283	0.721-2.281	0.397	1.405	0.760-2.598	0.279
Lymph node stage	negative *vs.* positive	2.167	0.980-4.796	0.056	2.001	0.864-4.637	0.106
Grade	G1 *vs.* G2 *vs.* G3	1.286	0.756-2.186	0.353	1.273	0.722-2.246	0.404
Histological type	IDC *vs.* ILC *vs.* Others	0.742	0.422-1.306	0.301	0.651	0.345-1.228	0.185
Combined grouping	others *vs.* PELP1, Ki-67 double high	2.020	1.022-3.990	0.043	2.380	1.138-4.978	0.021

Abbreviations: LN, lymph node; DFS, disease-free survival; OS, overall survival; HR, hazard ratio; 95 % CL, 95 % confidence interval

Note: ^a^for the variable, data for two cases are unavailable from medical records

## Discussion

Although previous studies have shown that PELP1 functions as an oncogene that is deregulated in breast cancer [14, 16], little is known about the prognostic significance of PELP1 in TNBC. Our study provided three new insights into the predictive role of PELP1 in TNBC: first, high PELP1 protein expression is correlated with positive lymph node status in TNBC; second, for the TNBC patients presenting with small tumor size or high Ki-67 LI, high PELP1 protein expression in the tumor is associated with a poor outcome; third, double high expression of PELP1 and Ki-67 in TNBC is associated with poorer patient outcomes, and was found to be an independent prognostic factor.

In our study, PELP1 was exclusively nuclear in localization. This result is consistent with recent immunohistochemical studies using commercially available antibodies against PELP1 in a variety of tissues [12, 14, 17]. However, PELP1 has been suggested to be involved in both the nuclear-initiated and membrane-initiated action of estrogen, and earlier IHC studies performed at the MD Anderson Cancer Center also reported PELP1 to have extensive cytoplasmic location in a panel of tumor tissues [9, 11, 18, 19]. A possible explanation for this discrepancy may lie in the different antibodies against PELP1 used in these studies. Of note, the antibody used in the IHC studies from the MD Anderson Cancer Center was developed by the local laboratory, and was raised by challenging a rabbit with a 19-mer peptide encoding 558–576 amino acids residues in the center of PELP1 [18]. However, most commercial antibodies against PELP1, including the antibody used in this study (Bethyl Laboratory; Cat. IHC-00013), as well as that used in the Habashy et al. study (Novus Biologicals; Cat.NB100-1749) [14], were raised to recognize the epitopes between residues 1000–1050 in the C-terminal of PELP1, which has been identified as a region for PELP1 interaction with cytoplasmic proteins, such as the p85 subunit of phosphatidylinositol 3-kinase (PI3K) [18, 20]. Thus, the epitope recognized by these commercially available antibodies might be masked when PELP1 is localized in the cytoplasm, and leave only nuclear immunostaining detectable by IHC.

H-score is the gold standard for quantifying nuclear immunoreactivity of IHC specimens because it takes into account both immunointensity and immunoreactivity, allowing an accurate approximation of the protein content. Additionally, previous studies have used the H-score approach to quantify PELP1 immunoreactivity [14], which led us to adopt a similar approach for our quantification of immunostaining of PELP1. PELP1 protein expression in our TNBC cohort (54.3 % ≥ 170) was significantly higher compared with that of unselected breast cancers (13.5 % ≥ 170) in the Habashy et al. study [14]. Although assessment of strong PELP1 expression in the TNBC group is not available from the Habashy et al. study, the positive correlation of PELP1 with expression of basal cytokeratin (CK-14, CK-5/6) and the negative correlation with ER and PR in unselected breast cancer reported in that study suggested a relatively higher expression of PELP1 in TNBC [14].

In our TNBC cohort, PELP1 protein expression showed positive correlations with lymph node stage. Although no association between PELP1 expression and lymph node stage was found, the expression of PELP1 demonstrated to be positively correlated with distant metastasis in the Habashy et al. study [14]. Several studies have suggested PELP1 may play an important role in metastasis of tumors including breast [21], ovarian [22], endometrial [23] and prostate cancer [24]. PELP1 had been reported to interact with several proteins involved in cell adhesion and extracellular matrix remodeling, such as Src kinase, PI3K, Integrin-linked kinase 1, and Metastasis-associated protein 1 [21]. In ER -negative breast cancer, deregulated PELP1 modulated the transcription of genes involved in epithelial-to-mesenchymal transition (EMT) and enhanced the activity of matrix metalloproteinases, thereby promoting tumor invasion and metastasis. In line with these findings, PELP1 knockdown reduced the *in vivo* metastatic potential of ER-negative breast cancer cells and significantly reduced lung metastasis in an *in vivo* xenograft assay [10]. Thus, our finding of a correlation between PELP1 expression and lymph node metastasis is consistent with previous studies that documented the oncogenic and pro-metastatic properties of PELP1 and may explain the poor prognosis observed in PELP1-expressing, highly proliferative TNBC tumors.

The prognostic significance of PELP1 varies among carcinomas, and seems dependent on the cellular context. Early studies proposed PELP1 expression as a predictor of poor outcome in patients with multiple types of carcinomas, including breast [14], endometrial [11], colorectal [13], and prostate cancers [24]. However, the most recent study examining PELP1 as a prognostic marker found it was associated with favorable prognosis in ERβ-positive ovarian cancer [12]. Overall, the divergent results between these studies suggest that PELP1 may have different prognostic impact in settings of different tumors or possibly within different subgroups of the same tumor. In our study, PELP1 did not show a significant independent association with either OS or DFS in TNBC patients, though patients with higher PELP1 expression demonstrated a trend of reduced DFS and OS, compared with those with less PELP1 expression (*p* = 0.089 for DFS, *p* = 0.074 for OS, log rank test).

As TNBC is inherently a heterogeneous subgroup of breast cancer, we considered the possibility that further sub-division of TNBC may be necessary to fully appreciate any potential role of PELP1 [25]. Ki-67, an indicator of cell proliferation, has been previously used to further sub-classify TNBC, and breast cancer patients with a Ki-67 LI >14 % were considered to have poorer outcomes [15, 26]. In this study, by combining PELP1 status with other clinicopathological variables to create a biological marker for predicting prognosis of TNBC, we found that patients with double high PELP1/Ki-67 expression (PELP1 H-score ≥170 and Ki-67 LI >14 %) had significantly reduced OS and DFS, in comparison with the other subgroups. Multivariable analysis also indicated that high expression of both PELP1 and Ki-67 in TNBC was an independent prognostic factor, with an adjusted HR of 2.020 for recurrence (95 % CL, 1.022–3.990; *p* = 0.043) and 2.380 for death (95 % CL, 1.138–4.978; *p* = 0.021). Despite the limited sample size in the present study, our results still suggest that combining PELP1 and Ki-67 expression as a biological marker may enhance the prognostic sensitivity of the two biomarkers in TNBC.

In addition to its potential as a prognostic marker, PELP1 expression has also been suggested as a candidate therapeutic target for treating hormone-related cancers [22, 27]. In previous *in vitro* studies, reduction of PELP1 expression by RNA interference (RNAi) exhibited a substantial inhibitory effect on proliferation, invasion, and therapeutic resistance of tumor cells [21, 28–30]. However, the challenges, such as off-target effects, toxicity and safe delivery methods, associated with the clinical application of RNAi-based therapeutics remain. Therefore, at this juncture, RNAi is not yet considered a viable therapeutic approach [31]. However, recent studies have indicated that this may change. For example, a team from The University of Texas reported the development of a novel, stable, non-toxic, small molecule peptidomimetic, which can disrupt the specific interaction between PELP1 and the androgen receptor and demonstrates a functional abrogation of androgen-induced proliferation of prostate cancer cells [32]. This finding suggests a promising future for PELP1-targeted therapy, but whether this small molecule peptidomimetic will also work against breast cancer, especially in TNBC cases, still needs further investigation.

## Conclusions

Despite the limitation of a small sample size used in this study, our findings indicate that considering PELP1 and Ki-67 expression systemically in TNBC will enhance the prognostic sensitivity of the two biomarkers, as high expression of both PELP1 and Ki-67 in tumors is an independent prognostic factor predicting poorer outcome of patients with TNBC. Furthermore, this finding suggests that PELP1 may be a valuable therapeutic target for TNBC in the future.

## Abbreviations

AC: Adriamycin/Cyclophosphamide
AC-T: Adriamycin/Cyclophosphamide-Taxol
DFS: Disease-free survival
DOGH: Daqing Oilfield General Hospital
EMT: Epithelial-mesenchymal transition
ERα: Estrogen receptor alpha
FAHHMU: The Fifth Affiliated Hospital of Harbin Medical University
HER-2: Human epidermal growth factor receptor 2
IDC: Invasive ductal carcinoma
IHC: Immunohistochemistry
ILC: Invasive lobular carcinoma
Ki-67 LI: Ki-67 label index
LN: Lymph node
OS: Overall survival
PELP1: Proline, glutamic acid, leucine rich protein 1
PI3K: Phosphatidylinositol 3-kinase
PR: Progesterone receptor
TNBC: Triple-negative breast cancer
